# *Flavobacterium psychrophilum* vaccine development: a difficult task

**DOI:** 10.1111/1751-7915.12099

**Published:** 2014-07-23

**Authors:** Esther Gómez, Jessica Méndez, Desirée Cascales, José A Guijarro

**Affiliations:** Área de Microbiología, Departamento de Biología Funcional, Facultad de Medicina, IUBA, Universidad de Oviedo33006, Oviedo, Asturias, Spain

## Abstract

Bacterial cold water disease (BCWD) is a globally distributed freshwater fish disease caused by the Gram-negative bacterium *Flavobacterium* *psychrophilum*. It is a particularly devastating infection in fry salmonids and may lead to high levels of mortality. In spite of its economic impact on fish farms, neither the biology of the bacterium nor the bacterium–host interactions are well understood. This review provides a synopsis of the major problems related to critical remaining questions about research into the use of vaccines against *F*. *psychrophilum* and the development of a commercial vaccine against this disease. Studies using sera from convalescent rainbow trout have shown the antigenic properties of different proteins such as OmpH, OmpA and FspA, as well as low and high molecular mass lipopolysaccharide of *F. psychrophilum*, which are potential candidates for subunit vaccines. Inactivated *F. psychrophilum* bacterins have been successfully tested as vaccines under laboratory conditions by both immersion and intraperitoneal routes. However, the efficacy and the practical usefulness of these preparations still have to be proved. The use of attenuated and wild-type strains to immunize fish showed that these systems offer high levels of protection. Nevertheless, their application clashes with the regulations for environmental protection in many countries. In conclusion, protective vaccines against BCWD are theoretically possible, but substantial efforts still have to be made in order to permit the development of a commercial vaccine.

## Introduction

*Flavobacterium psychrophilum* is the etiological agent of bacterial cold water disease (BCWD). Juvenile rainbow trout *(Oncorhynchus mykiss)* are particularly affected, and, in this case, the disease is called rainbow trout fry syndrome.

The disease affecting rainbow trout and Coho salmon (*Oncorhynchus kisutch*) was known only in North America (Borg, 1948). The first cases in Europe were reported in France and Germany in mid-1980s (von Weis, 1987; Bernardet *et al*., 1988; Bernardet and Kerouault, 1989). Shortly after this, more cases of infection in Coho salmon and ayu (*Plecoglossus altivelis*) were reported in Japan (Wakabayashi *et al*., 1991). Subsequently, the disease appeared in all parts of the world (Bernardet and Bowman, 2006), becoming one of the main causes of economic losses in salmonid aquaculture.

Although external signs of infection are variable depending on both the fish species and their development stage, in larger fish they typically consist of skin erosion and muscle degeneration followed by progressive tissue necrosis (Cipriano and Holt, 2005; Barnes and Brown, 2011). In addition, septicaemia can develop, without any of these characteristic external signs mainly in fry (Cipriano and Holt, 2005; Barnes and Brown, 2011).

The disease appears most frequently when water temperatures range between 10°C and 14°C (Borg, 1960). As there is no commercial vaccine, treatment with antibiotics is the only means for its eradication. However, resistance to these compounds is increasing (Cipriano and Holt, 2005), and the number of them available for the treatment of BCWD is limited. These are the main reasons for interest in the development of an effective vaccine against BCWD.

*Flavobacterium psychrophilum* is considered a ‘fastidious’ bacterium because it is difficult to culture, isolate and manipulate, but, in spite of this, the need for an effective vaccine has motivated research into this organism. Thus, in the last few years, the development of genetic techniques (Alvarez *et al*., 2004; Perez-Pascual *et al*., 2011; Gomez *et al*., 2012) and culture media (Alvarez and Guijarro, 2007; Perez-Pascual *et al*., 2010) together with the availability of the complete genome sequence of the bacterium (Duchaud *et al*., 2007) have facilitated its study and, in particular, that of its virulence determinants. In this respect, some factors associated with pathogenesis have been described. Among these are extracellular proteases (Bertolini *et al*., 1994; Ostland *et al*., 2000), an iron acquisition system (Alvarez *et al*., 2008), an adhesin (Nematollahi *et al*., 2003), haemolytic activities (Lorenzen and Olesen, 1997; Lorenzen *et al*., 1997; Moller *et al*., 2003) and a thiol: disulphide oxidoreductase (Alvarez *et al*., 2006). Besides, putative virulence genes have been identified in the genome analysis of this bacterium (Duchaud *et al*., 2007). Additionally, different studies in bacterial surface structures have identified major antigens such as lipopolysaccharide (LPS) O-antigens (MacLean *et al*., 2001) and surface proteins (Crump *et al*., 2001; 2005; Massias *et al*., 2004; Dumetz *et al*., 2007), which open new perspectives for vaccine development. However, mechanisms enabling the bacterium to evade the fish immune system have been described for *F. psychrophilum*; it was, for example, able to suppress the non-specific humoral defence mechanisms (Siwicki *et al*., 2004). Indeed, viable bacteria have been found in spleen phagocytes, where they are protected against lysozyme activity, complement and exposure to the immune system (Decostere *et al*., 2000). The bacterium must have additional mechanisms of protection against the complement system because high complement activity in rainbow trout serum was unable to reduce the number of *F. psychrophilum* cells (Wiklund and Dalsgaard, 2002). Therefore, specific immune responses must exist in order to avoid the dispersion of the infection.

To obtain an effective vaccine for *F. psychrophilum*, besides the understanding of virulence factors and the identification of putative antigens, it is essential to understand certain other aspects of the infection process, such as the route by which the bacterium enters the fish, serotype variability and the immune response of the fish after both natural and experimental infections. All these questions, especially the latter, are the main subjects that will be analysed in this review.

## Why is the development of a vaccine a difficult task?

There are still a number of fundamental points that have to be resolved before a vaccine for BCWD can be developed and commercialized. A key question for vaccine development is the identification of the point of entry of the bacterium. In the case of *F. psychrophilum*, these data have not yet been established, although most authors suggest that BCWD could be a water-borne infection, and the bacterium could penetrate fish via damaged skin or gills (Dalsgaard, 1993). Infected fish could also act as a reservoir in a carrier state, constantly shedding bacteria into the water and transmitting the bacteria by horizontal transfer (Madsen and Dalsgaard, 1999; Madetoja *et al*., 2000; Madsen *et al*., 2005). Additionally, some authors described the presence of the bacterium in healthy fish, as part of the microbiota of the fins, gills and opercula of adult salmonids (Nematollahi *et al*., 2003). When the fish immune system is not in optimal conditions, the bacterium might cause infection. The presence of the bacterium in the perivitellin space of the eggs, milt and ovarian fluids has been reported, and vertical transmission of *F. psychrophilum* via intra-ovum infection has already been suggested by several authors (Brown *et al*., 1997; Izumi and Wakabayashi, 1997; Rangdale *et al*., 1997; Vatsos *et al*., 2001; 2006; Cipriano, 2005). Therefore, prevention measures such as iodophor treatment of the eggs are not totally effective because the bacterium is protected within the perivitellin space (Kumagai *et al*., 2000), which represents a significant source of infection from the earliest life stages. Nevertheless, it should be taken into account that some authors (Madsen *et al*., 2005; Madsen and Dalsgaard, 2008) did not find the bacterium inside eggs, but still found that disinfection could be an effective method for the elimination of the bacteria.

Bearing all this in mind, vaccination against BCWD should be carried out as early as possible. However, a major problem remains: the bacterium may cause the young fish problems when they are as small as 0.2–0.5 g and consequently, their immune system is not fully developed, which compromises the usefulness of the vaccine. In salmonid fish, the onset of adaptive immunity occurs when they weigh from 0.5 to 2.5 g, according to the results found in vaccination by immersion against *Yersinia ruckeri* (Ellis, 1988) and *Vibrio anguillarum* (Johnson *et al*., 1982; Tatner and Horne, 1983). At these weights, there is another question to consider for vaccination may only be possible by immersion or in-feed administration because the small size of the immature fish makes other routes of administration unviable for economic and practical reasons. However, administration of the vaccine by these routes does not produce a long-term immune response, and if the fish were vaccinated early, they probably would not have enough protection. The injection route induces the strongest and longest immune response; however, it should only be used with fish weighing over 15 g (Hastein *et al*., 2005), and the vaccine needs to be combined with an adjuvant to induce a long-term immunological response. This adjuvant has a double function: it improves the presentation of the antigen to the immune cells and keeps it within the fish tissues, thus increasing the length of protection. However, adjuvants cause undesirable side effects such as reactions in the abdominal cavity, so they have to be previously tested to minimize these problems (Hastein *et al*., 2005). It should be borne in mind that most of the fish vaccination studies cited in this review do not clearly state the role of the innate immune system in protection. In this sense, it would be useful for studies on fish vaccination to distinguish between the effect of adjuvants and other immunostimulants on the activation of the innate immune system and, on the other hand, the adaptive immune responses to the specific antigens.

In addition, reproducibility of experimental *F. psychrophilum* vaccination is not yet well established, and there is no standardized vaccination model (Decostere *et al*., 2000). Immersion trials are the most difficult to reproduce, and experimental conditions have to be established in order to obtain reliable results. By contrast, although reproducible intraperitoneal infection models have been established (Madsen and Dalsgaard, 1999; Garcia *et al*., 2000), the intraperitoneal route bypasses the natural barrier of the fish to external bacterial threats and is therefore not the best model to analyse vaccine effectiveness.

Another factor that should be studied in depth before designing an effective vaccine is the serotyping within the bacterial species. One of the first serotype classifications (Lorenzen and Olesen, 1997) identified only three serotypes when Danish isolates were compared with others from the rest of Europe (Table 1). These serotypes were ‘Th’, which was predominant among Danish isolates; ‘Fd’, a minority serotype, and ‘Fp^T^’, found in isolates that did not come from clinical outbreaks. Izumi and Wakabayashi (1999) defined a similar number of serotypes (Table 1). However, the authors of both classifications pointed out that besides the three defined serotypes, there might be others that were host specific. In fact, Mata *et al*., identified seven host-specific serotypes in 2002 (Table 1), and Izumi and colleagues (2003) detected a novel serotype isolated from amago (*Oncorhynchus masou rhodurus*). Therefore, differences in serotype should be determined, and cross protection experiments have to be carried out in order to design a vaccine with broad-spectrum effectiveness. The development of increasingly sophisticated molecular techniques made it possible to distinguish different fish species-associated ribotypes (Chakroun *et al*., 1998). In addition, by multilocus sequence typing analysis on isolates from 10 different host species and four continents, Nicolas and colleagues (2008) showed that there were some clonal complexes with worldwide geographic distribution showing a high level of association with particular fish species. Thus, it seems that there is a correlation among host and particular serotypes, ribotypes and also clonal complexes. This means an additional difficulty for the creation of a vaccine for preventing infection in a wide range of *F. psychrophilum* strains.

**Table 1:** Comparison of the most representative serological classifications of *F. psychrophilum*

Host	Serotype classification		
	Mata and colleagues (2002)	Lorenzen and Olesen (1997)	Izumi and Wakabayashi (1999)
Salmon	1	Fp^T^	O1
Trout	2a	Fd	
Trout	2b	Th-2	
Trout	3	Th-1	O3
Eel	4		
Carp	5		
Tench	6		
Ayu	7		O2

Finally, as a minor consideration, technical complications may arise when large amounts of bacteria or particular antigens have to be produced because of the handling difficulties and the fastidious nature of *F. psychrophilum*.

## Potential vaccine targets and vaccination assays performed with *F. psychrophilum*

The investigation of virulence factors in *F. psychrophilum* has led to the definition of potential vaccine candidates, from proteins with immunogenic properties, to attenuated strains resulting from mutations in genes related to the infection process. This review includes the principal vaccination trials with subunit, inactivated, attenuated and even wild-type *F. psychrophilum* strains, as well as studies in which potential vaccine targets are suggested for further investigations.

### Potential subunit vaccines

Studies on the surface characteristics of *F. psychrophilum* have led to the identification of several antigens that may be potential vaccine candidates.

In 2001, Crump and colleagues, in an extensive antigen characterization of several strains, identified three immunogenic cell surface molecules of *F. psychrophilum* that elicited a strong immunological response in fish. Rainbow trout convalescent antiserum, obtained from fish that had survived challenge with *F. psychrophilum* 259–93, recognized, apart from a 20 kDa protein, two molecular mass classes of LPS: a low-molecular-mass LPS antigen of 16 kDa present in the outer layer and a high-molecular-mass LPS (∼100 kDa) containing O antigen, which was abundant in culture supernatants (Crump *et al*., 2001). The authors concluded that these could be potential vaccine candidates. Two years later, Merle and colleagues (2003) also identified (by Western blotting) about 20 major cell envelope antigens which reacted with rabbit antibodies produced against membrane proteins. Among them, the glycoprotein P60 was one of the predominant antigens in the cell envelope of *F. psychrophilum*. This protein was selectively extracted with Triton X-100 and easily purified, and it was proposed as one of the clear candidates for use as a subunit vaccine. Dumetz and colleagues (2007) identified this P60 membrane glycoprotein, called OmpA, as one of the immunodominant antigens of the bacterium. Anti-OmpA antibodies were able to inhibit *F. psychrophilum* growth, showing that OmpA is readily accessible on the cell surface and could be important to the physiology of the bacterium.

Another surface-located *Flavobacterium*-specific protein antigen (FspA) strongly recognized by convalescent rainbow trout sera was characterized by Crump and colleagues (2005). This FspA protein was identified by two-dimensional gel electrophoresis and immunoblotting, and further extensively characterized by nanospray-tandem mass spectrometry and peptide sequencing. This antigen was also expressed in *Escherichia coli* as a fusion protein able to retain its immunoreactivity, representing a well-characterized subunit vaccine candidate against BCWD.

More recently, Sudheesh and colleagues (2007) compared proteins from cellular and extracellular products of a virulent (CSF-259-93) and non-virulent (ATCC 49418) strains of *F. psychrophilum* in order to identify antigens associated with either virulence or host immunity. By Western blot analysis combined with two-dimensional gel electrophoresis, several immunogenic proteins reacting with rainbow trout anti-CSF-259-93 serum and shared by both strains were identified. However, at least 15 immunogenic proteins seemed to be unique for the virulent strain, and four proteins were exclusive for the non-virulent one. By liquid chromatography-mass spectrometry analysis, some of them were identified, and two heat shock proteins, HSP60 and HSP70 were found to be of particular interest. These cellular proteins were expressed in both virulent and non-virulent strains of *F. psychrophilum* and were highly reactive against trout antiserum, indicating that they were indeed immunodominant antigens of the bacteria. However, Plant and colleagues (2009) showed, in an experimental challenge, that vaccination of rainbow trout by recombinant and DNA vaccines based on these proteins resulted in no protection against *F. psychrophilum*.

Apart from all the above-described components representing possible candidates for a *F. psychrophilum* subunit vaccine, Dumetz and colleagues (2008) defined the outer membrane subproteome of the bacterium in order to identify the dominant antigens targeted by the rainbow trout immune system during infection. An immunoproteomic approach using antibodies from BCWD-convalescent rainbow trout allowed the identification of 25 immunoreactive antigens. These included not only previously characterized antigens such as OmpA and FspA, but also newly described antigen proteins and lipoproteins of unknown function, providing a useful reservoir from which to design new vaccines. Recently, LaFrentz and colleagues (2011) performed an immunoproteomic analysis of *F. psychrophilum*. They used two-dimensional polyacrylamide gel electrophoresis and Western blotting with sera from fish immunized with high- and mid-molecular mass fraction proteins of the bacterium. Mass spectrometry was used to determine the protein identity. Fifteen immunogenic proteins were positively identified following Mascot searches in the *F. psychrophilum* genome. Results included outer membrane protein OmpA (P60), trigger factor, ClpB, elongation factor G, gliding motility protein GldN and a conserved hypothetical protein.

Most of these described *F. psychrophilum* antigens were not tested as potential vaccines in fish. Nevertheless, some *F. psychrophilum* preparations have been shown to increase the relative protection of fish against bacterial infection. Thus, Rahman and colleagues (2002) tested a vaccine preparation consisting of the outer membrane fraction of *F. psychrophilum*, obtained from a Sarkosyl cell lysate after ultracentrifugation, and including proteins with a range of molecular masses from 25 to 120 kDa. This preparation was injected intraperitoneally into juvenile rainbow trout and ayu, which produced high antibody titres and resulted in a high relative percent survival (RPS), significantly greater than those in fish immunized with a formalin-killed bacterin (FKB). The increased protection was probably due to the outer membrane proteins and LPS present in a highly enriched preparation. Therefore, this kind of preparation could be useful for further studies using immersion or in-feed administration of vaccine.

LaFrentz and colleagues (2004) evaluated the protective immune response that a specific range of molecular mass proteins from *F. psychrophilum*, when emulsified with Freund’s complete adjuvant (FCA), elicited in rainbow trout. The authors demonstrated that protective antigens of *F. psychrophilum* were primarily within the molecular weight range of 60–200 kDa and could involve LPS O-polysaccharide chains and/or proteins. Immunization using the 70–100 kDa region resulted in nearly complete fish protection with a mean RPS of 94% at the lowest challenge dose (6.25 × 10^6^ colony-forming units per fish). Western blot analysis using sera from fish immunized with the 70–100 kDa region showed that high molecular weight proteins and carbohydrates are recognized by serum antibodies. Initially, it was hypothesized that the carbohydrates corresponded to high molecular mass LPS containing O-polysaccharide (LaFrentz *et al*., 2004). However, some years later, LaFrentz and colleagues (2007) found that the antigens were not LPS, but the components of the glycocalyx of *F. psychrophilum*. These antigens may be involved in eliciting the highly protective immune response. Therefore, they are important candidates for consideration during the development of vaccines against BCWD.

Another candidate for the development of a vaccine is the 18 kDa outer membrane-associated OmpH-like protein identified by Massias and colleagues (2004) and, subsequently, characterized by Dumetz and colleagues (2006). This protein might have different roles, including a chaperone periplasmic function. Vaccination trials by intraperitoneal injection using a fraction highly enriched with the OmpH-like protein with or without FCA induced significant protective immunity in fish, with 54.1% and 88.5% RPS respectively. This protection is probably the result of the relatively high titres of antibodies produced against the flavobacterial OmpH-like protein, which also has antimicrobial activities (Dumetz *et al*., 2006).

Crump and colleagues (2007) implemented a new strategy to find a subunit vaccine for *F. psychrophilum*. A recombinant vaccine in *E. coli* was generated to avoid the difficulties of handling *F. psychrophilum*. A highly expressed 166-amino-acid fusion protein in *E. coli*, homologous to the bacterial ribosomal protein L10, reacted with convalescent rainbow trout serum. When this chimaera, adjuvanted with FCA, was injected intraperitoneally into rainbow trout, an 82% RPS was obtained, indicating a high level of protection against virulent *F. psychrophilum* challenge. However, it should be taken into account that control fish injected with FCA adjuvant reached a 47% RPS, while saline injected fish presented values below 15%, which clearly demonstrates that the adjuvant has a pronounced effect on the level of protection, probably though the innate immune system. This adjuvant effect should be always assessed in fish vaccination studies because it seems that it could be quantitatively important in protection. Although it is not expected that a ribosomal protein located inside the bacterium could be a main inducer of the adaptive immune system, there are other described cases in *Brucella abortus* (Oliveira *et al*., 1996; Ribeiro *et al*., 2002) and *Leishmania* species (Iborra *et al*., 2005; Stober *et al*., 2006).

Finally, Plant and colleagues (2011) cloned and purified three recombinant proteins to be used as vaccines in rainbow trout: elongation factor-Tu, SufB Fe-S assembly protein and ATP synthase β of *F. psychrophilum*. Unfortunately, the results indicated that they are not suitable vaccine candidates, at least when administered alone as purified proteins via intraperitoneal injection.

### Potential inactivated cell vaccines

Inactivated bacterial whole-cell vaccines are the most commonly used to induce immunity because of their safety and effectiveness when they are administered via injection. There are different methods for obtaining non-viable cells without causing cellular lysis, such as formalin or heat inactivation. From the studies, a significant level of fish immunization can be inferred when adjuvanted-inactivated *F. psychrophilum* cells were used as vaccines. Consequently, they should be considered as good candidates for an inactivated vaccine.

In juvenile rainbow trout sera and mucosae, LaFrentz and colleagues (2002) tested antibody responses after immunizing fish with an average weight of 10 g by the intraperitoneal route using *F. psychrophilum* FKB preparations, together with FCA. In these conditions, a strong serum antibody response was found after 6 weeks. However, 9 weeks was needed to detect significant mucosal antibody titres. In addition, following a 12-week immunization, subcutaneous injection challenge with live *F. psychrophilum* cells was performed. The results showed that 83% RPS protective immunity was conferred to fish immunized with FKB + FCA. Interestingly, a 51% RPS was obtained when saline solution + FCA was used. These results showed that the stimulation of non-specific immune factors increase the immune response. However, specific antibodies were necessary to provide nearly complete protection. Nevertheless, immersion vaccination trials did not confer any protection.

The efficacy of *F. psychrophilum* FKB was also evaluated in juvenile ayu with an average weight 0.5 g, but in this case, the vaccine was orally administered (Kondo *et al*., 2003). Juvenile ayu were fed with dry pellets mixed with the FKB, either every day for 2 weeks or on 5 days over 2 weeks. At 3 and 7 weeks after vaccination, experimental immersion challenges were performed. The results showed, in both challenge tests, a significant increase in survival rates in orally vaccinated fish compared with control fish. However, the daily-vaccinated fish group had lower survival rates than the other group with 5 days administration over 2 weeks. The authors pointed out that this could be due to an immunological tolerance probably caused by formalin, as Joosten and colleagues (1995) and Patrie-Hanson and Ainsworth (1999) reported for *V. anguillarum* in carp (*Cyprinus carpio*) and *Edwardsiella ictaluri* in channel catfish (*Ictalurus punctatus*) respectively.

Heat- or formalin-inactivated *F. psychrophilum* of Fd or Th serotypes, adjuvanted with mineral oil, were intraperitoneally injected into rainbow trout with an average weight of 50 g, in order to test the immune response generated (Madetoja *et al*., 2006). After fish vaccination, antibody titres were significantly higher in vaccinated fish sera than in non-vaccinated ones. In addition, vaccinated fish, when challenged against *F. psychrophilum* under laboratory conditions, showed a significantly higher survival rate compared with non-vaccinated fish (Madetoja *et al*., 2006). However, in the case of skin mucus, specific antibody production was not increased (Madetoja *et al*., 2006).

Interestingly, orally administered *F. psychrophilum* FKB from logarithmic phase cultures were able to induce immunity in rainbow trout of 1.6 g average weight (Aoki *et al*., 2007). However, when FKB for vaccination were obtained from stationary phase cultures, immunization was significantly lower. In logarithmic phase cultures, *F. psychrophilum* cells had many membrane vesicles on the surface that were released into the medium during the stationary phase. The presence of these vesicles in the vaccine preparation seemed to play an important role because in rainbow trout immunized with stationary phase cells combined with a membrane vesicle-rich supernatant, the protection resulted in an RPS of 94–100%, although immunization with membrane vesicle-rich supernatant alone resulted in no protection against *F. psychrophilum* infection (Aoki *et al*., 2007). Although the nature of the vesicle components was not analysed by the authors, they suggested that a combination of these membrane vesicles, which probably consist of outer membrane lipids, outer membrane proteins and soluble periplasmic components, with FCB was the best choice to obtain high protection levels against the disease.

An intraperitoneal injection vaccine has been developed by Fredriksen and colleagues (2013a). Whole *F. psychrophilum* formalin-inactivated cells of an isolated strain cross binding the Th and Fd serotypes were used as a vaccine in a water-in-oil formulation. Intramuscular infection challenges in rainbow trout (average weight 36.6 g) using a highly virulent *F. psychrophilum* strain demonstrated a high level of protection against BCWD (Fredriksen *et al*., 2013a).

Similarly, Fredriksen and colleagues (2013b), showed high protection levels (RPS higher than 77.5%) in rainbow trout (average weight 33.1 g), with both divalent and multivalent (named FLAVO IPN and FLAVO AVM6 respectively) intraperitoneally administered vaccines containing whole-cell antigens of *F. psychrophilum*.

### Potential attenuated vaccines

Live attenuated strains of *F. psychrophilum* may offer the advantage of enhancing the stimulation of the immune system for long periods, whereas the subunit and whole cell-inactivated vaccines have a shorter-lasting effect. However, safety issues as well as environmental aspects must be considered before releasing this kind of vaccine. These risks include reversion to virulence, the spreading of antibiotic resistance genes, exchange of genetic information with other bacteria, changes in host tropism, etc. Therefore, this type of vaccine has the disadvantage of having to be submitted to strict controls and regulations, thus making its application difficult in certain countries. Therefore, up to now only a few examples of this kind of vaccine can be found.

Alvarez and colleagues (2008) obtained an attenuated *F. psychrophilum* mutant in which the gene coding for the *ExbD2* protein was disrupted. This protein is part of the TonB complex and is involved in iron uptake. Intramuscular injection of this mutant followed by challenge experiments with a virulent *F. psychrophilum* strain showed a significant protective immune response in rainbow trout fry. Indeed, 6 weeks after intramuscular immunization using the attenuated strain, an RPS value of 81.8% was obtained. It should be pointed out that some vaccinated fish developed skin lesions after challenge, but, in contrast to the normal progression of the disease, these lesions gradually healed in a few days, at least on visual examination, probably as a consequence of the immune response generated. This healing phenomenon was also observed by LaFrentz and colleagues (2008).

In vaccination studies with attenuated strains, LaFrentz and colleagues (2008) found that a selected rifampicin-resistant *F. psychrophilum* strain, called 259-93B.17, was highly attenuated. This mutant presented five differentially expressed proteins in relation to the wild-type strain, but the role of these proteins, as well as the mechanisms involved in its antibiotic resistance, were still unknown. Rainbow trout immunization by intraperitoneal injection with the 259-93B.17 strain resulted in significant protection against *F. psychrophilum* wild-type strain and produced high specific antibody titres at 8 and 15 weeks after immunization. However, a more relevant result is that fish that had undergone immersion-immunization with the 259-93B.17 strain developed protective immune responses at 10 weeks post-immunization. Consequently, this strain may be useful as an attenuated vaccine against BCWD.

Further studies identified a mutation in the *rpoB* gene, which encodes the β-subunit of the RNA polymerase of the 259-93B.17 attenuated strain (Gliniewicz *et al*., 2012). In this study, eight proteins specific to the wild-type strain and six specific to the attenuated strain were identified (Gliniewicz *et al*., 2012). Among them, a putative antigen (FP1493) was distinguished by Western blotting. This antigen was later expressed as a recombinant protein and tested as a subunit vaccine. Unfortunately, it did not confer protection against the parental strain. So other differentially expressed and immunoreactive proteins should be evaluated for their effectiveness as subunit vaccines.

### Potential wild-type based vaccines

The use of wild-type strains of the bacterium for developing potential vaccines has been evaluated. Thus, rainbow trout fries were immersed with two pathogenic Danish isolates at the same time and, after a 26-day post-intraperitoneal challenge with the more pathogenic isolate, RPS values were 88.2% and 25% of the fry were positive in specific antibody titres (Lorenzen *et al*., 2010). However, despite their apparently being very effective in generating immunity, the use of wild-type-based vaccines leads to the risk of virulent strains being released into the environment.

A different vaccination approach was undertaken by Sugahara and Eguchi (2012). Instead of studying the effects of an inactivated vaccine, they investigated the effectiveness of warmed water as a treatment against *F. psychrophilum* in ayu fish. To this end, after 1, 6 or 24 h immersion in a live bacterial suspension, ayu were treated with warmed water (28°C) for three days. Fourteen days after immersion, high antibody titres against *F. psychrophilum* and cumulative mortalities of 36%, 30% and 18% were obtained for warmed water-treated fish after 1, 6 and 24 h immersion respectively. These values were significantly lower than those obtained in control fish (90%). From the results, they concluded that warmed water treatment could cure BCWD in ayu. The authors considered that warmed water probably acted as an inactivation treatment that led *F. psychophilum* cells into the viable but non-culturable state inside the fish organs, having a vaccinating effect (Sugahara *et al*., 2010). However, this method should only be applied to naturally infected fish as a treatment, but not as a means of vaccination by immersion with live cells because of the risk of environmental contamination with these strains. Additionally, it could not be applied to cold-water fish because of the temperature needed for this procedure.

### Passive immunization

Anti-*F. psychrophilum* sera from either convalescent or immunized adult trout or goat were injected intraperitoneally into rainbow trout fry. Then they were challenged by subcutaneous injection with a virulent strain of *F. psychrophilum*. Higher RPS rates were obtained when fry were administered with sera from immunized adult rainbow trout instead of with sera from convalescent fish. However, goat anti-*F. psychrophilum* serum did not confer protection to fry. Taking into account these results, the authors suggested that antibodies are not the only factors playing a relevant role in conferring protection against *F. psychrophilum* (LaFrentz *et al*., 2003).

## Conclusions and future approaches

The readers of this review may observe that during the last 20 years, multiple attempts have been made to obtain a commercial vaccine for preventing BCWD. A significant number of antigens have been identified that confer immunization in particular conditions. In addition, cross protection between Fd and Th isolated serotypes has been demonstrated, suggesting that some of the major antigens could be shared by both serotypes. In the same way, inactivated vaccines were able to induce a good immunological response. However, most of these experiments were carried out using antigen administration routes that are impractical for field application. The antigenic components of *F. psychrophilum* are well defined, but a procedure for an efficient immersion or oral administration system must still be developed. Attenuated- and virulent-based vaccines are seen to be promising candidates for obtaining a strong immune response induction. However, the use of this kind of vaccine means some environmental and safety risks have to be managed, and most countries, particularly those in the European Union, have restrictive regulations on their use. Moreover, the use of bacteria as delivery vectors for recombinant heterologous antigens implies the construction of recombinant strains and their release into nature. This situates attenuated and live recombinant vaccines in the debate, and there is not yet a consensus as to whether they should be used.

Another important field in fish vaccination is the development of immunostimulants and adjuvants that are administrated before, with or after the vaccines and which could amplify the immune response by increasing the antibody titres in sera and mucus without having inappropriate effects on the fish.

Despite the important advances that have been made in the last few years in relation to different aspects of vaccination against *F. psychrophilum*, new strategies and initiatives are needed in order to develop a definitive commercial vaccine. Studies on oral vaccine administration based on the stimulation of the gut-associated lymphoid system of fish should be conducted. This involves the use and development of an encapsulation method to protect antigens against degradation in the foregut. Nevertheless, it should be established, according to previous experiences, that this procedure may result in a low immune response and an inadequate duration of protection. Concerning immersion vaccination trials, emphasis should be put on the factors influencing the uptake of a particular antigen by fish in order to develop mechanisms to improve this uptake. Consequently, the most appropriate immersion times, water temperatures, antigen concentrations and the tissues participating in the uptake should be established. The lack of normalized values for these parameters is a serious obstacle to further progress, and it is probable that vaccination against *F. psychrophilum* will not be achieved successfully without addressing these issues.
